# Clinicopathological and prognostic correlations of HER3 expression and its degradation regulators, NEDD4–1 and NRDP1, in primary breast cancer

**DOI:** 10.1186/s12885-018-4917-1

**Published:** 2018-10-26

**Authors:** Satu Luhtala, Synnöve Staff, Anne Kallioniemi, Minna Tanner, Jorma Isola

**Affiliations:** 1 BioMediTech Institute and Faculty of Medicine and Life Sciences, University of Tampere, Tampere, Arvo Ylpön katu 34, 33520 Tampere, Finland; 2 Department of Obstetrics and Gynecology, Tampere University Hospital, Tampere, Finland; 3 Department of Oncology, Tampere University Hospital, Tampere, Finland

**Keywords:** HER3, ErbB3, NEDD4–1, NRDP1, FLRF, RNF41, Prognostic biomarker, Survival, Breast cancer

## Abstract

**Background:**

Human epidermal growth factor receptor HER3 (ErbB3), especially in association with its relative HER2 (ErbB2), is known as a key oncogene in breast tumour biology. Nonetheless, the prognostic relevance of HER3 remains controversial. NEDD4–1 and NRDP1 are signalling molecules closely related to the degradation of HER3 via ubiquitination. NEDD4–1 and NRDP1 have been reported to contribute to HER3-mediated signalling by regulating its localization and cell membrane retention. We studied correlations between HER3, NEDD4–1, and NRDP1 protein expression and their association with tumour histopathological characteristics and clinical outcomes.

**Methods:**

The prevalence of immunohistochemically detectable expression profiles of HER3 (*n* = 177), NEDD4–1 (*n* = 145), and NRDP1 (*n* = 145) proteins was studied in primary breast carcinomas on archival formalin-fixed paraffin-embedded (FFPE) samples. Clinicopathological correlations were determined statistically using Pearson’s Chi-Square test. The Kaplan-Meier method, log-rank test (Mantel-Cox), and Cox regression analysis were utilized for survival analysis.

**Results:**

HER3 protein was expressed in breast carcinomas without association with *HER2* gene amplification status. Absence or low HER3 expression correlated with clinically aggressive features, such as triple-negative breast cancer (TNBC) phenotype, basal cell origin (cytokeratin 5/14 expression combined with ER negativity), large tumour size, and positive lymph node status. Low total HER3 expression was prognostic for shorter recurrence-free survival time in *HER2*-amplified breast cancer (*p* = 0.004, *p* = 0.020 in univariate and multivariate analyses, respectively). The majority (82.8%) of breast cancers demonstrated NEDD4–1 protein expression - while only a minor proportion (8.3%) of carcinomas expressed NRDP1. NEDD4–1 and NRDP1 expression were not associated with clinical outcomes in *HER2*-amplified breast cancer, irrespective of adjuvant trastuzumab therapy.

**Conclusions:**

Low HER3 expression is suggested to be a valuable prognostic biomarker to predict recurrence in *HER2*-amplified breast cancer. Neither NEDD4–1 nor NRDP1 demonstrated relevance in prognostics or in the subclassification of *HER2*-amplified breast carcinomas.

**Electronic supplementary material:**

The online version of this article (10.1186/s12885-018-4917-1) contains supplementary material, which is available to authorized users.

## Background

Human epidermal growth factor receptor HER3 (ErbB3), a cell membrane-associated protein encoded by the *ERBB3* gene, is a promising target for cancer therapy, especially in HER2-positive (carrying *ERBB2*/*HER2* gene amplification) breast carcinoma [1]. Both HER3 and HER2 belong to a family of epidermal growth factor receptor (EGFR, HER) tyrosine kinases that activate after receptor dimerization. This culminates in the initiation of signal transduction pathways that markedly regulate cellular viability [1]. When catalytically defective, HER3 is unable to homodimerize and orchestrate its own activation [2, 3]. HER3 is known to interact most preferably with its structurally homologous relative HER2 once bound with its ligand heregulin (HRG), also called neuregulin-1 [4–6]. Heterodimerization between HER2 and HER3 induces subsequent PI3K/AKT and Ras/Raf/MAPK signalling cascades [7]. The presence of HER3, as an allosteric activator, is required to maintain active HER2-mediated signalling [8, 9], and aberrantly intensified HER2-HER3 signalling is hence critically associated with breast carcinogenesis and tumour cell proliferation [4, 10–12].

HER3 protein overexpression has been shown to commonly co-occur with *HER2* gene amplification and HER2 overexpression, therefore, HER3 is thought to contribute markedly to the pathogenesis of *HER2*-amplified breast cancer subtype [4, 13, 14]. The co-expression of HER2 and HER3 proteins [15, 16] and abundance of HER2-HER3 heterodimers in situ have also been associated with adverse clinical outcomes in breast cancer [17–19]. The formation of HER2-HER3 heterodimers also inhibits HER3 downregulation [20]. Due to the close interaction between HER2 and HER3, dual inhibitory therapy is preferred and clinically relevant treatment for carcinomas with altered HER2 signalling [8, 11, 21]. In addition to HER2-positive breast carcinomas, therapeutic targeting of HER3 receptors has been suggested also in the treatment of HER3-dependent, HER2-negative breast cancers to prevent cell growth-promoting signalling triggered by intensified HER3-HER1 heterodimerization [22]. Several HER3-targeting molecules have been developed as therapeutics, and many of them are currently being tested in clinical trials [23, 24].

After a careful survey of the literature, it appears that the prognostic value of HER3 expression (at the protein or mRNA level) in breast cancer is controversial (Table 1). Overexpressed HER3 is mostly associated with a worse survival [16, 25–35], but conflicting results have also been published [36–40]. Many studies did not find any demonstrable relationships between HER3 and patient survival [15, 41–53]. Studies focusing on HER3 specifically in *HER2*-amplified breast cancer [16, 25, 26, 29, 31, 32, 37, 38, 41, 44, 45, 48, 49, 52, 54] have not drawn conclusive results either. Interestingly, HER3 activation has been implicated as a molecular mechanism inducing inherent or acquired de novo resistance to anti-HER2 therapy [19, 31, 55, 56]. Continuous inhibition of HER2 signalling may lead to compensatory HER3 activation, which results from heterodimerization between HER3 and its alternative dimerization partner HER1 [57, 58].

**Table 1:** Literature review of studies relating to HER3 prognostics in human breast cancer

Publication by	Laboratory Methodology	Cohort Characteristics	Prognostic Implications
*Takada* et al. [91]	IHC (RTJ2)	met-HER2+ BCA (*n* = 29), TPD	↓ Low HER3 expression was associated with shortened PFS
*Adamczyk* et al. [25]	IHC (SP71)	HER2+ BCA (*n* = 97), Adj.T	↑ High HER3 expression (only with concurrent PTEN negativity) was associated with shorten MFS
*Duchnowska* et al. [44]	VeraTag assay	HER2+ BCA (*n* = 189), Adj.T	- No correlation between HER3 expression and OS in advanced stage HER2 + BCA
*Nishimura* et al. [54]	VeraTag assay	met-HER2+ BCA (*n* = 47), T	- HER3 expression did not has any influence on PFS in trastuzumab-refractory advanced HER2 + BCA
*Koutras* et al. [39]	qRT-PCR	BCA (*n* = 663, HER2 + BCA *n* = 143)	↓ Low HER3 mRNA (only with concurrently high EGFR, high HER2, low HER4 mRNA) was associated with worse DFS
*Baselga* et al. [38]	qRT-PCR^*^, IHC^**^ (DAK-H3-IC)	HER2+ BCA (*n* = 740^*^/497^**^), Adj.T	↓ High HER3 mRNA was associated with better prognosis in metastatic HER2 + BCA
*Berghoff* et al. [16]	IHC (DAK-H3-IC)	met-BCA (*n* = 110, met-HER2 + BCA *n* = 34)	↑ High HER3 expression was associated with shorter OS in initially metastatic HER2 + BCA subgroup
*Park* et al. [31]	IHC (DAK-H3-IC)	met-HER2+ BCA (*n* = 125), T	↑ High HER3 expression was associated with worse PFS in initially metastatic HER2 + BCA
*Bae* et al. [26]	IHC (DAK-H3-IC)	HR-BCA (*n* = 886, HER2 + BCA *n* = 221)	↑ High HER3 expression was associated with poorer DFS in HER2 + BCA subgroup and poorer DFS and OS in TNBC
*Czopek* et al. [48]	IHC (DAK-H3-IC)	HER2+ BCA (*n* = 35)	- No correlation between HER3 expression and DFS or OS
*Lipton* et al. [29]	VeraTag assay	met-HER2+ BCA (*n* = 89), T	↑ High HER3 expression was associated with shorter PFS in initially metastatic HER2 + BCA
*Gori* et al. [41]	IHC (RTJ1)	met-HER2+ BCA (*n* = 61), T	- HER3 was not significantly associated with clinical outcome in initially metastatic HER2 + BCA
*Han* et al. [37]	VeraTag assay	met-HER2+ BCA (*n* = 50), T	↓ High HER3 expression was related to longer TTP in advanced HER2 + BCA
*Larsen* et al. [43]	IHC (DAK-H3-IC)	ER+ BCA (*n* = 1062)	- HER3 expression did not shown any association to DFS
*Chiu* et al. [27]	IHC (Ab-10 pAb)	BCA (*n* = 3123)	↑ High HER3 expression was associated with decreased BCSS
*Yonemori* et al. [45]	IHC (DAK-H3-IC)	HER2+ BCA (*n* = 44), neoAdj.T	- HER3 expression did not significantly correlate with pCR
*Giltnane* et al. [28]	AQUA	BCA (*n* = 550)	↑ High HER3 expression was associated with decreased survival
*Haas* et al. [42]	IHC (SGP1)	HER2- BCA (*n* = 171)	- No prognostic value for HER3
*Sassen* et al. [50]	IHC (5A12), FISH	BCA (*n* = 173)	- No prognostic value for HER3 expression, *HER3* gene amplification was related to decreased DFS
*Giuliani* et al. [52]	IHC (RTJ1)	met-HER2+ BCA (*n* = 103), T	- No prognostic value for HER3
*Lee* et al. [36]	IHC (pAb)	BCA (*n* = 378)	↓ High HER3 expression correlated with longer DFS
*Bianchi* et al. [53]	IHC (RTJ1)	BCA (*n* = 145)	- No prognostic value for HER3 expression singly, but high co-expression of HER2/3/4 predicted worse prognosis
*Fuchs* et al. [34]	IHC (C-17 pAb)	BCA (*n* = 48)	↑ High HER3 expression singly and in co-expression with high HER1 and HER2 was associated with poor prognosis
*Robinson* et al. [32]	IHC (polyclonal)	met-HER2+ BCA (*n* = 104), T	↑ High HER3 expression was associated with worse OS
*Wiseman* et al. [33]	IHC (2-18C9)	BCA (*n* = 242)	↑ High HER3 expression independently and with high HER1 and/or HER2 was associated with decreased DSS
*Abd El-Rehim* et al. [15]	IHC (RTJ1)	BCA (*n* = 1499)	- No prognostic value for HER3 singly, but in co-expression with high HER2 predicted unfavorable DFS and OS
*Smith* et al. [49]	IHC	met-HER2+ BCA (*n* = 77), T	- No prognostic value for HER3
*Bièche* et al. [35]	qRT-PCR	BCA (*n* = 130)	↑ High HER3 mRNA was associated with shorten RFS
*Witton* et al. [30]	IHC (H3.105.5)	BCA (*n* = 220)	↑ High HER3 expression was associated with reduced BCSS survival
*Suo* et al. [47]	IHC (sc-415), RT-PCR	BCA (*n* = 100)	- High HER3 expression was predictive for reduced DFS or BCSS only in co-overexpression with HER2 or HER1 + HER2
*Pawlowski* et al. [40]	qRT-PCR	BCA (*n* = 365)	↓ Elevated HER3 mRNA expression was associated with a better prognosis in terms of OS, but did not relate to RFS
*Travis* et al. [46]	IHC (RTJ1)	BCA (*n* = 346), met-BCA (*n* = 145)	- No prognostic value for HER3 expression neither in primary nor metastatic breast cancer
*Lemoine* et al. [51]	IHC (49.3 pAb)	BCA (*n* = 195)	- No demonstrable relationship between HER3 expression and survival

*Abbreviations*: Adj.T = adjuvant trastuzumab therapy; BCA = primary breast cancer; BCSS = breast cancer-specific survival; DFS = disease-free survival; DSS = disease-specific survival; ER+ BCA = oestrogen receptor-positive breast cancer; HER2- BCA = HER2-negative breast cancer; HER2+ BCA = HER2-positive primary breast cancer; HR- BCA = hormone receptor-negative breast cancer; IHC = immunohistochemistry (antibody clone); met- = breast cancer diagnosed at advanced stage; MFS = metastasis-free survival; neoAdj.T = neoadjuvant trastuzumab therapy; n = number of patients being determined for HER3 status and followed for survival; OS = overall survival; pAb = polyclonal antibody; PFS = progression-free survival; pCR = pathologically complete response; qRT-PCR = quantitative reverse transcription polymerase chain reaction; RFS = recurrence-free survival; T = trastuzumab therapy after metastasis; TNBC = triple-negative breast cancer; TPD = trastuzumab, pertuzumab, docetaxel regimen; TTP = time to progression; ↑ = high HER3 mRNA or protein expression associated with worse clinical outcome; ↓ = low HER3 mRNA or protein expression associated with worse clinical outcome

The exact mechanisms behind aberrant HER3 protein expression have not been fully elucidated [13]. Unlike HER2, HER3 does not undergo gene amplification during breast carcinogenesis [16, 59, 60]. Cancer-related *ERBB3* mutations are relatively uncommon, except for colon and gastric carcinomas [59, 61]. One hypothesis is that excessive cellular HER3 expression may be due to defects in downstream signalling mechanisms that regulate HER3 membrane trafficking [13]. Aberrant expression of HER3 degradation regulators may lead to an abnormal accumulation or deficit of membrane-bound HER3 receptors, consequently influencing HER3 signalling efficiency. Here, we studied the expression of two proteins, NEDD4–1 (*ne*ural precursor cell expressed *d*evelopmentally *d*ownregulated *4–1*) and NRDP1 (*n*euregulin *r*eceptor *d*egradation *p*rotein *1*, also known as FLRF and RNF41), which are known to be necessary for HER receptor quantity control [62]. NEDD4–1 [63] and NRDP1 [64–67] are both E3 ubiquitin protein ligases suggested to crucially downregulate HER3 and its subcellular localization by mediating HER3 receptors to degradation via the ubiquitin-proteasome-pathway. Defects in ubiquitination are critical and lead to aberrant receptor activity and signalling [68]. Hypothetically, HER3 overexpression may be associated with the concurrent absence of its ubiquitination regulators, NEDD4–1 and NRDP1.

Low NEDD4–1 expression due to *NEDD4–1* knockdown has been demonstrated to activate HER3 and increase cancer cell proliferation in vivo and in vitro [63]. Conversely, NEDD4–1 overexpression has resulted in decreased HER3 expression and increased HER3 ubiquitination [63]. Aberrant expression of NEDD4–1 has been implicated in the pathogenesis and adverse prognosis of several human malignancies [69–72]. Despite the frequent overexpression in breast cancer [73, 74], the prognostic value of NEDD4–1 remains unclear in the clinical context.

NRDP1, in turn, is less frequently overexpressed than NEDD4–1 in breast carcinoma [75, 76]. NRDP1 overexpression has been shown to cause a decrease in HER3 expression and an inhibition of breast cancer cell growth in vitro [75]. Conversely, a loss of NRDP1 followed by *NRDP1* knockdown suppressed HRG-induced HER3 ubiquitination and degradation in MCF7 breast cancer cells [64]. An inverse correlation between NRDP1 and HER3 expression in situ has been demonstrated in breast tumours derived from *ERBB2* transgenic mice [75] and in human breast carcinomas [76]. The prognostic and clinical significance of NRDP1 remains unknown. In the current study, we studied the association between HER3, NEDD4–1, and NRDP1 protein expression, clinicopathological characteristics and clinical outcomes in primary breast cancer, especially in the *HER2*-amplified subtype.

## Methods

### Clinical sample material

Two separate archival sample collections of formalin-fixed paraffin-embedded (FFPE) primary breast carcinomas were used for biomarker analyses conducted in compliance with the REMARK guidelines [77]. The first sample collection, “the BCA cohort”, consisted of 308 primary, invasive breast carcinomas that were diagnosed in the area served by Tampere University Hospital between 1990 and 1999. Of these carcinomas, 47 (15.3%) were characterized as HER2-positive based on HER2 protein overexpression. Lobular carcinomas were overrepresented in this cohort compared to the overall prevalence of this type of carcinoma (Table 2). This sample set was prepared as tissue microarray (TMA) sections and was originally established for another study, which has been described in more detail in publications by Korhonen et al. [78, 79]. Primary treatment for patients was conducted according to the existing clinical practice: surgery, post-operative radiotherapy, adjuvant cytotoxic chemotherapy (mostly CMF) and endocrine therapy (Table 3).

**Table 2:** Clinicopathological characteristics of primary breast cancer patients in BCA cohort and HER2+ BCA cohort

Characteristic	*n*	BCA cohort, n (%)	*n*	*HER2*-amplified BCA cohort, n (%)
Follow-up period for RFS (range)		*Mean 10.4 yr. (1 mo.-22 yr.)*		*Mean 5.3 yr. (1 mo.-9 yr.)*
Age (range)	308	*Median 61 yr. (32–93 yr.)*	177	*Median 60 yr. (29–91 yr.)*
< 50 years		64 (20.8)		36 (20.3)
≥ 50 years		244 (79.2)		141 (79.7)
HER2 status	308		177	
Positive		47 (15.3)		177 (100.0)
Negative		261 (84.7)		0 (0.0)
ER status	307		177	
Positive (≥10%)		248 (80.8)		113 (63.8)
Negative (< 10%)		59 (19.2)		64 (36.2)
PR status	307		177	
Positive (≥10%)		201 (65.5)		74 (41.8)
Negative (< 10%)		106 (34.5)		103 (58.2)
Triple negativity	307		177	
TNBC (HER2−/ER-/PR-)		30 (9.8)		0 (0.0)
No TNBC		277 (90.2)		177 (100.0)
Histological grade	232		174	
I-II		179 (77.2)		41 (23.6)
III		53 (22.8)		133 (76.4)
Ki67 proliferation index	230		177	
Low (< 20%)		165 (71.7)		33 (18.6)
High (≥20%)		65 (28.3)		144 (81.4)
Histological type	304		168	
Ductal		173 (56.9)		156 (92.9)
Lobular		131 (43.1)		12 (7.1)
Tumour size	177		142	
< 2 cm		57 (32.2)		68 (47.9)
≥ 2 cm		120 (67.8)		74 (52.1)
Tumour size	308		172	
pT1-pT2		282 (91.6)		161 (93.6)
pT3-pT4		26 (8.4)		11 (6.4)
Lymph nodal spread	286		169	
Positive pN+		114 (39.9)		73 (43.2)
Negative pN0		172 (60.1)		96 (56.8)

Number of patient cases with available data (*n*) for each character is marked within the columns

**Table 3:** Primary treatments of patients in BCA and HER2+ BCA study cohorts

Primary treatment	BCA cohort (*n* = 308)		*HER2*-amplified BCA cohort (*n* = 177)	
	*n*	*%*	*n*	*%*
Breast surgery				
Mastectomy (*ablation*)	161	52.4	101	57.1
Conservative surgery (*resection*)	146	47.6	72	40.7
No operation			3	0.6
Unknown	1			
Post-operative radiotherapy	198	65.3	110	62.1
No	105	34.7	67	37.9
Unknown	5			
Adjuvant endocrine therapy	97	32.1	104	58.8
No	205	67.9	73	41.2
Unknown	6			
Adjuvant chemotherapy	40	13.4	133	75.1
No	259	86.6	44	24.9
Unknown	9			
Adjuvant trastuzumab			82	46.3
No	308	100.0	95	53.7

The other sample collection, specified as the “HER2+ BCA cohort”, consisted exclusively of 177 *HER2*-amplified invasive breast carcinomas diagnosed during the years 2003–2007 in the Pirkanmaa Hospital District. The status of hormone receptors, oestrogen receptor (ER) and progesterone receptor (PR), *HER2* gene amplification, and Ki67 proliferation index were determined during the diagnostic procedure, and related data were retrieved from the clinical records. *HER2* gene amplification status was previously determined by the chromogenic in situ hybridization (CISH) technique. This sample set was prepared as whole tissue sections. Approximately half (*n* = 82) of the carcinomas, primarily patients diagnosed after June 2005, were treated with conventional chemotherapy combined with adjuvant trastuzumab during 9-wk schema as a first-line therapy [80] for primary disease. The remaining patients (*n* = 95) did not receive any adjuvant HER2-targeted therapy for primary disease. In addition to surgery and adjuvant cytotoxic chemotherapy (mostly consisting of taxanes, CEF), post-operative radiotherapy and adjuvant endocrine therapy were given when necessary (Table 3).

Samples were selected for the current study according to the following inclusion criteria: availability of representative tumour tissue (FFPE), adequate pathological characterization, and clinical follow-up data. Clinicopathological data and follow-up information were collected, retrospectively. The mean follow-up period for recurrence-free survival (RFS) in the HER2+ BCA cohort was 5.3 years (range: 1 month to 9 years) and 10.4 years (range: 1 month to 22 years) for the BCA cohort. NEDD4–1 and NRDP1 expression was studied in a smaller fraction of the HER2+ BCA cohort representing available *HER2*-amplified cases (*n* = 145). Table 2 describes the clinicopathological characteristics of the study cohorts.

### Immunohistochemical stainings

For immunohistochemistry (IHC), serial four-μm-thick sections were cut from FFPE sample blocks and mounted on Super Frost Plus® slides followed by deparaffinization and dehydration. Heat-induced epitope retrieval (HIER) was performed in TE buffer (50 mM Tris 1 mM EDTA, pH 9) at 98 °C for 15 min. To determine HER3 protein expression, we used the optimized IHC staining protocol described in our earlier study [81]. We used a mouse monoclonal (clone DAK-H3-IC) antibody against the human HER3 protein at a dilution of 1:100. The expression of basal epithelium cytokeratins 5 and 14 was determined using the same IHC protocol with an antibody cocktail composed of anti-human mouse monoclonal antibodies CK14 (clone LL002) and CK5 (clone XM26), both diluted at 1:150. Ki-67 expression was determined similarly in BCA cohort samples with mouse monoclonal Ki-67 antibody (clone BS4) at a dilution of 1:100.

For NEDD4–1 IHC, we used rabbit polyclonal anti-NEDD4 WW2 domain antibody (dilution 1:750) to detect NEDD4–1 proteins. Bright Vision+ Poly-HRP-Anti-mouse/rabbit IgG kit (ImmunoLogic, AD Duiven, the Netherlands) and 3,3′-diaminobenzidine tetrahydrochloride DAB-2V kit (Nichirei Biosciences Inc., Tsukiji, Chuo-ku, Tokyo, Japan) were used for the detection of immunoreactivity according to manufacturers’ instructions. To detect the NRDP1 protein, we used rabbit polyclonal FLRF/RNF41 antibody (dilution 1:3000), EnVision™ FLEX High pH HRP and EnVision™ FLEX DAB+ reagents (Dako, Glostrup, Denmark), according to manufacturers’ protocols. After staining, slides were counterstained with Mayer’s Hematoxylin (Oy FF-Chemicals Ab, Haukipudas, Finland) with 1:4 addition of 2% copper sulfate to intensify the DAB reaction. Slides were then dehydrated, cleared with xylene and sealed with DePeX mountant.

All staining reactions were conducted using the LabVision™ Autostainer 480S platform. As positive control samples, we used human FFPE tissues known to express the specified proteins: normal prostate ductal cells for HER3 [82], kidney proximal tubule cells for NEDD4–1 [83], testicular cells in seminiferous ducts and mononuclear blood cells for NRDP1 [84]. A negative staining control was prepared by omitting and replacing the primary antibody with diluent reagent and was included in each staining batch. An additional file 1 and Table 4 present detailed information on antibodies and IHC-staining protocols used in the current study.

**Table 4:** Details of antibodies used in the IHC-protocols of the current study

Antibody	Host species	Catalog No.	Clonality	Dilution	Manufacturer/distributor
Anti-Human HER3	Mouse	M7297	DAK-H3-IC	1:100	DAKO A/S, Glostrup, Denmark
FLRF/RNF41 Antibody	Rabbit	A300-049A	polyclonal	1:3000	Bethyl Laboratories, Inc., Montgomery, Texas, USA
Anti-Nedd4, WW2 domain	Rabbit	#07–049	polyclonal	1:750	Merck KGaA, Darmstadt, Germany
Cytokeratin 5 Antibody	Mouse	NCL-L-CK5	XM26	1:150	Leica Biosystems Newcastle Ltd., Newcastle Upon Tyne, UK
Cytokeratin 14 Antibody	Mouse	NCL-L-LL0022	LL0022	1:150	Leica Biosystems, Newcastle Ltd., Newcastle Upon Tyne, UK
Anti-human Ki67	Mouse	BSH-7302	BS4	1:100	Nordic BioSite AB, Täby, Sweden

### Microscopic analysis and interpretation of immunoreactivity

Samples stained for HER3, NEDD4–1 and NRDP1 were scanned with SlideStrider (Jilab Inc., Tampere, Finland) into digital images that were examined virtually with JVSview JPEG2000 [85] and SlideVantage 1.2 (Jilab Inc., Tampere, Finland) viewer applications. The ImmunoRatio 2.5 application was used for automated cell counting of distinct cancer cells with nuclear immunoreactivity [86]. Staining patterns were analysed within the invasive cancerous tissue area displaying the most intense brown DAB reaction (region of interest, ROI).

For HER3 appearance, both membranous and cytoplasmic staining reactions were inspected on a computer screen. Samples were classified according to the staining intensity and proportion of specifically stained cancer cells as previously described [81]. Briefly, HER3 staining localized to the cancer cell outer membrane was considered ‘membranous’ and was scored according to the following criteria: (0) absent/low staining (< 10% of cells), (1+) intermediate circumferential staining (10–30% of cells) and (2+) strong circumferential staining (> 30% of cells). The staining reaction observed in the cancer cell cytoplasm was considered ‘cytoplasmic’ and was categorized as (0) no/faint staining, (1+) overall low-intensity staining, and (2+) prevalent high-intensity staining covering most of the cancer cells. Score 1+ was set as a threshold to define HER3 positivity both for membranous and cytoplasmic staining. Total HER3 staining was designated as negative for cases with low (0/1+) membranous staining concurrently with low (0/1+) cytoplasmic staining and as positive for cases with high (2+) membranous and/or (2+) cytoplasmic staining.

The NEDD4–1 protein expression pattern was analysed by scoring the staining intensity as follows: 0 (no staining), 1+ (weak), 2+ (moderate), and 3+ (strong). Samples with scores < 3+ were seen as NEDD4–1 negative ‘low expressing’ and samples with score 3+ as NEDD4–1 positive ‘high expressing’. Overall, the NEDD4–1 staining pattern in cancerous areas was homogenous, and therefore, the percentage of stained cells was not evaluated.

NRDP1 staining was analysed by applying a scoring system presented in a study by Jiao et al. [76]. We analysed nuclear and cytoplasmic staining separately. Staining intensity was scored accordingly: 0 (no staining), 1 (weak), 2 (moderate), and 3 (strong). Based on the percentage of stained cancer cell nuclei, samples were classified as 0 (< 1%), 1 (1–24%), 2 (25–49%), 3 (50–74%), and 4 (75–100%). The grades were then multiplied to determine a score for low and high nuclear expression. Cases with scores ≤3 were defined as ‘low expressing’ and those with scores ≥4 as ‘high expressing’. Cytoplasmic NRDP1 expression was categorized as high if the staining intensity in the tumour cells was moderate or strong. Expression patterns of basal epithelium cytokeratins 5 and 14 and Ki67 protein were analysed with Olympus System Microscope BX43. Carcinomas were interpreted as positive for CK5 and CK14 expression if more than 20% of the malignant cells displayed clear cytoplasmic staining [87]. For Ki67 protein expression, we used a 20% cut-off value to determine low (< 20%) and high (≥20%) cell proliferation activity [86].

### Statistical analysis

All statistical data analyses were performed using IBM® SPSS® Statistics version 23 (IBM Corp.). Generally, *p*-values < 0.05 were considered statistically significant for any relationship being considered. Proportions among categorical variables were compared using Pearson’s Chi-Square test to determine clinicopathological correlations. Kaplan-Meier survival analysis and log-rank test (Mantel-Cox) were used to compare survival differences for each categorical variable. RFS time was chosen as the endpoint for the current study. To determine RFS, patients were followed from the date of surgery for initial diagnosis to the date of disease progression as local recurrence or distant metastasis. Patients who did not experience recurrence during the follow-up were censored at the time of death or last date of medical record inspection.

## Results

### HER3 protein expression in breast carcinomas

**Fig. 1:**
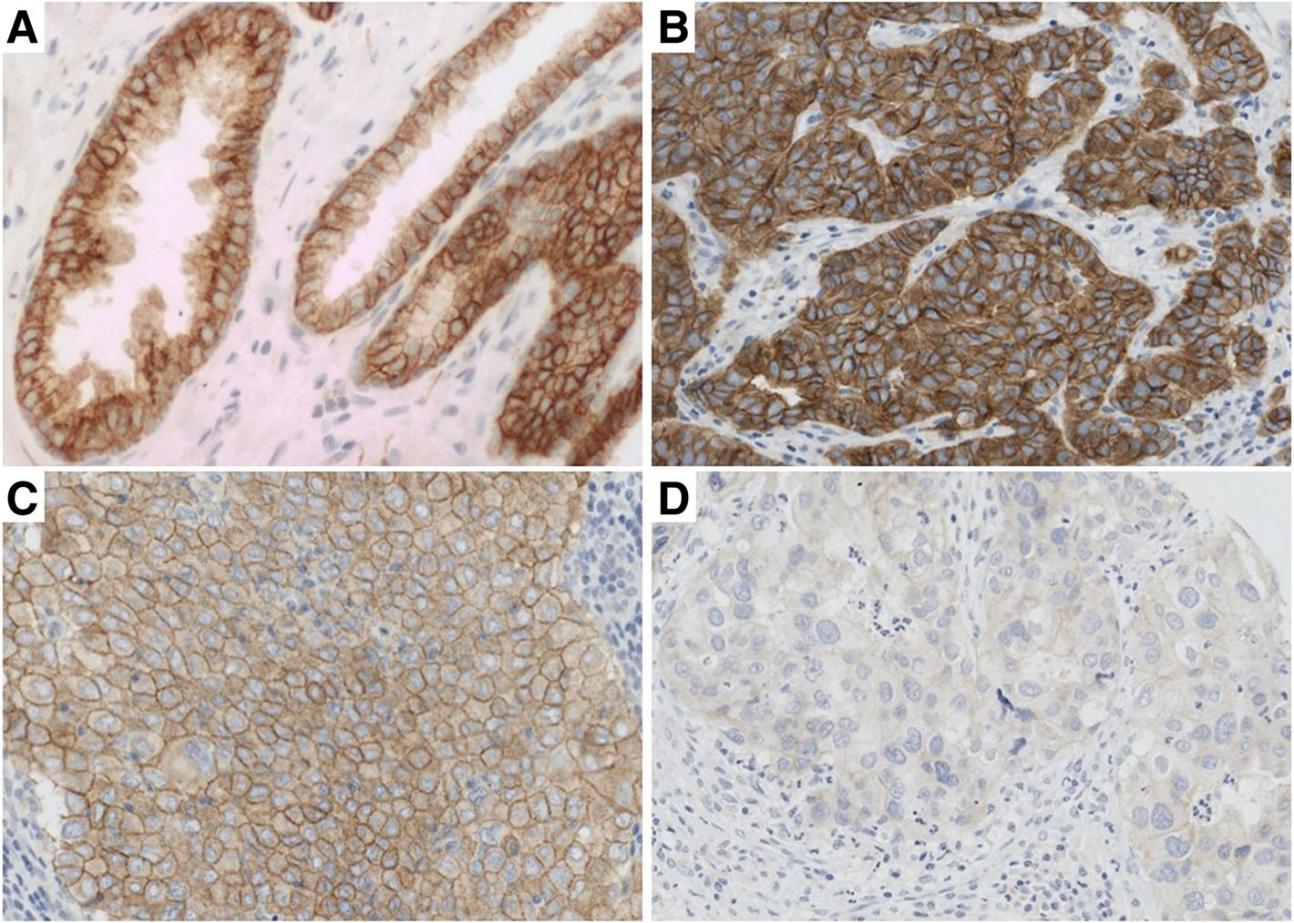
HER3 immunohistochemistry. **a** Positive control (prostate), **b** Concurrently high (score 3+) membranous and cytoplasmic HER3 expression (breast carcinoma), **c** High (score 3+) membranous HER3 expression with negative/low (score 0) cytoplasmic HER3 status, **d** Negative/low total cellular HER3 staining. Mayer's Hematoxylin used as a counterstain

In the BCA sample set consisting of HER2-positive and -negative breast carcinomas (BCA cohort), high membranous HER3 expression was observed in half of the cases (51.9%, 160 of 308). Nearly all (95.8%, 295 of 308) carcinomas showed HER3 protein expression localized in the cancer cell cytoplasm. When the total cellular HER3 expression pattern was evaluated, the majority (75.3%, 232 of 308) of carcinomas were classified as HER3-positive, ‘high total HER3 expressing’. One-fourth of the carcinomas (24.7%, 76 of 308) were determined to be HER3-negative, ‘low total HER3 expressing’. Figure 1 shows examples of membranous and cytoplasmic HER3 IHC staining patterns observed in the present study.

### HER3, NEDD4–1, and NRDP1 protein expression in *HER2*-amplified breast carcinomas

To determine whether HER3 protein expression is common in *HER2*-amplified breast cancer subtype, we also studied HER3 expression in the HER2+ BCA cohort established for this purpose. We noticed that 80.2% (142 of 177) of *HER2*-amplified breast carcinomas showed complete circumferential membrane staining for HER3. Cytoplasmic HER3 staining was more common, since only a small fraction (8.5%, 15 of 177) of these carcinomas were completely unstained. High total HER3 expression was demonstrated in 75.7% of cases (134 of 177), and one-fourth of carcinomas were designated as HER3-negative. Overall, HER3 protein was heterogeneously expressed within the cancerous areas represented in whole tissue sections. The HER3 staining pattern was, therefore, equally evaluated from the ROI showing the most intense DAB reaction (Fig. 1).

**Fig. 2:**
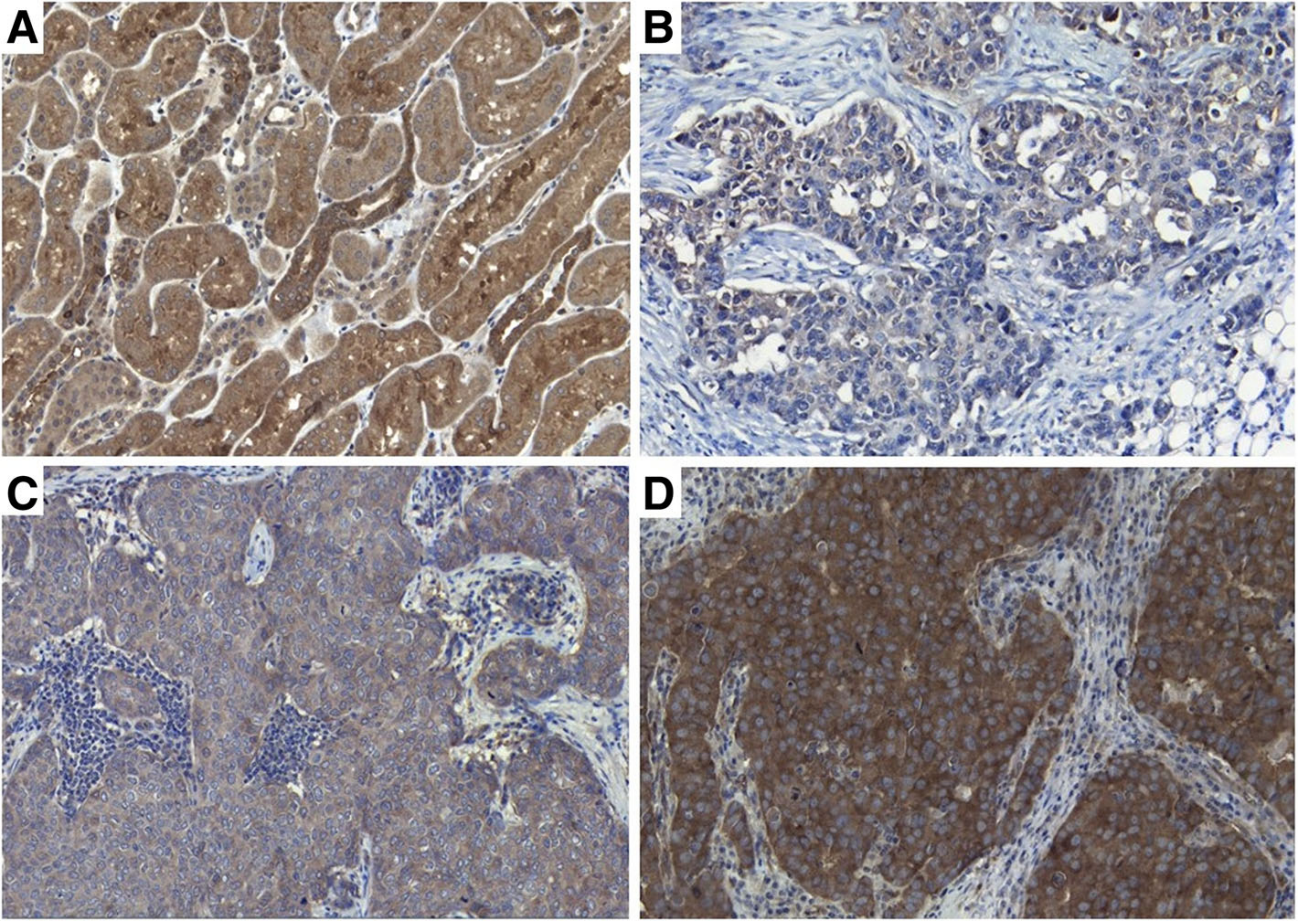
NEDD4–1 immunohistochemistry. **a** Positive control (kidney), **b** Negative/low NEDD4–1 expression (score 1+, breast carcinoma), **c** Moderate NEDD4–1 expression (score 2+, breast carcinoma), **d** High NEDD4–1 expression (score 3+, breast carcinoma). Mayer's Hematoxylin used as a counterstain

**Fig. 3:**
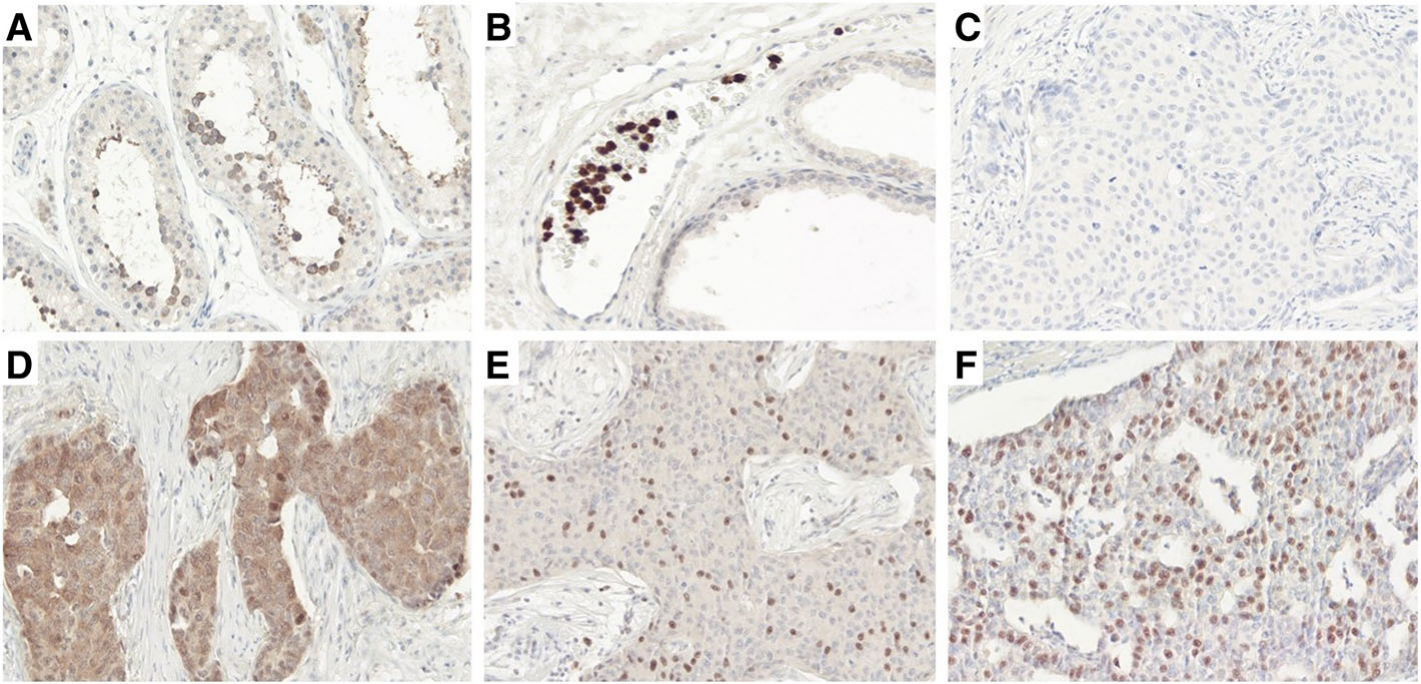
NRDP1 immunohistochemistry. **a** Positive control (testis, cells in seminiferous ducts), **b** Positive control (mononuclear blood cells), **c** Absent NRDP1 expression (breast carcinoma), **d** Cytoplasmic NRDP1 expression (breast carcinoma), **e** and **f** Nuclear NRDP1 expression (breast carcinoma). Mayer's Hematoxylin used as a counterstain

Next, we studied NEDD4–1 and NRDP1 protein expression in a cohort of *HER2*-amplified breast carcinomas. Most of the cases (82.8%, 120 of 145) demonstrated strong-to-moderate NEDD4–1 staining localized predominantly in the cytoplasmic region (Fig. 2). Approximately one-fifth (17.2%, 25 of 145) of the cases were categorized as NEDD4–1 low expression based on faint IHC staining reaction. The staining intensity and subcellular localization of NEDD4–1 protein were homogenous within the cancerous areas. Cells in histologically normal breast ducts were also positive for NEDD4–1. NRDP1 protein expression was uncommon in *HER2*-amplified breast carcinomas. NRDP1 localization in carcinoma cells was clearly nuclear or cytoplasmic (Fig. 3). The high presence of nuclear or cytoplasmic NRDP1 protein was observed in a minor proportion (8.3%, 12 of 145) of samples, while the majority of carcinomas (91.7%, 133 of 145) were classified as low for NRDP1 expression.

### Association of HER3, NEDD4–1 and NRDP1 with clinicopathological characteristics

In the BCA cohort, we noticed that HER3 protein expression was not dependent on HER2 status irrespective of its cellular localization (membranous *p* = 0.615, cytoplasmic *p* = 0.990, total *p* = 0.882). In addition, we found that low membranous HER3 protein expression was associated with an aggressive triple-negative breast cancer (TNBC) phenotype (*p* = 0.000), defined as concurrently negative ER, PR, and HER2 statuses. Similarly, negative PR receptor status alone (*p* = 0.002) and larger tumour size ≥2 cm (*p* = 0.003) were related to low membranous HER3. Cytoplasmic or total cellular HER3 expression were not associated with any particular clinicopathological characteristics (Table 5). HER3 expression was not related to neither cellular proliferation activity (Ki67) nor lymph nodal status. When the BCA cohort was analysed and stratified for HER2 status, we noticed that clinicopathological correlations were statistically significant only in HER2-negative carcinomas. In this group, low membranous HER3 expression was strongly associated with negative ER (*p* = 0.003) and negative PR (*p* = 0.002) statuses, high (III) grade (*p* = 0.008) and larger (≥2 cm) tumour size (*p* = 0.006).

**Table 5:** Associations between HER3 protein expression and clinicopathological characteristics

Characteristic	*BCA cohort HER2-amplified BCA cohort*																	
	HER3-m (%)		*p*	HER3-c (%)		*p*	HER3-t (%)		*p*	HER3-m (%)		*p*	HER3-c (%)		*p*	HER3-t (%)		*p*
	-	+		-	+		-	+		-	+		-	+		-	+	
HER2 status			0.615			0.990			0.882									
Negative	85.8	83.8		84.6	84.7		84.2	84.9										
Positive	14.2	16.2		15.4	15.3		15.8	15.1										
Estrogen receptor status			0.057			0.280			0.839			0.013*			0.376			0.104
Negative	23.6	15.1		30.8	18.7		18.4	19.5		54.3	31.7		46.7	35.2		46.5	32.8	
Positive	76.4	84.9		69.2	81.3		81.6	80.5		45.7	68.3		53.3	64.8		53.5	67.2	
Progesterone receptor status			0.002**			0.368			0.443			0.888			0.882			0.716
Negative	43.2	26.4		46.2	34.0		38.2	33.3		57.1	58.5		60.0	58.0		55.8	59.0	
Positive	56.8	73.6		53.8	66.0		61.8	66.7		42.9	41.5		40.0	42.0		44.2	41.0	
Triple-negativity (HER2-/ER-/PR-)			0.000***			0.099			0.798									
No	83.8	96.2		76.9	90.8		89.5	90.5										
Yes	16.2	3.8		23.1	9.2		10.5	9.5										
Histological grade			0.121			0.855			0.705			0.435			0.767			0.956
I-II	72.8	81.4		75.0	77.3		75.4	77.8		28.6	22.3		26.7	23.3		23.3	23.6	
III	27.2	18.6		25.0	22.7		24.6	22.2		71.4	77.7		73.3	76.7		76.7	76.4	
Ki-67 proliferation index			0.597			0.985			0.852			0.475			0.213			0.658
Low	73.4	70.2		71.4	71.7		72.7	71.4		22.9	17.6		6.7	19.8		20.9	17.9	
High	26.6	29.8		28.6	28.3		27.3	28.6		77.1	82.4		93.3	80.2		79.1	82.1	
Histological type			0.629			0.359			0.204			0.055			0.940			0.960
Ductal	55.5	58.2		69.2	56.4		63.2	54.8		85.3	94.8		93.3	92.8		92.7	92.9	
Lobular	44.5	41.8		30.8	43.6		36.8	45.2		14.7	5.2		6.7	7.2		7.3	7.1	
Lymph nodal status			0.531			0.637			0.716			0.232			0.169			0.035*
Negative pN0	58.3	61.9		66.7	59.9		62.0	59.5		65.7	54.5		40.0	58.4		42.9	61.4	
Positive pN+	41.7	38.1		33.3	40.1		38.0	40.5		34.3	45.5		60.0	41.6		57.1	38.6	
Tumor size (TNM stage)			0.840			0.921			0.781			0.173			0.001***			0.368
pT1-pT2	91.9	91.3		92.3	91.5		90.8	91.8		88.6	94.9		73.3	95.5		90.7	94.6	
pT3-pT4	8.1	8.7		7.7	8.5		9.2	8.2		11.4	5.1		26.7	4.5		9.3	5.4	
Tumor size (cm)			0.003**			0.834			0.357			0.643			0.014*			0.143
<2cm	21.6	42.7		28.6	32.4		26.7	34.1		51.7	46.9		15.4	51.2		37.1	51.4	
≥2cm	78.4	57.3		71.4	67.6		73.3	65.9		48.3	53.1		84.6	48.8		62.9	48.6	
Patient age at diagnosis			0.726			0.624			0.217			0.069			0.000***			0.156
<50 years	21.6	20.0		15.4	21.0		15.8	22.4		31.4	17.6		60.0	16.7		27.9	17.9	
≥50 years	78.4	80.0		84.6	79.0		84.2	77.6		68.6	82.4		40.0	83.3		72.1	82.1	
Cytokeratin 5/14 expression												0.583			0.561			0.006**
Negative										85.3	88.7		92.9	87.6		76.2	92.0	
Positive										14.7	11.3		7.1	12.4		23.8	8.0	
Basal phenotype (CK5/14+, ER-)												0.191			0.801			0.001***
No										85.3	92.5		92.9	90.8		78.6	95.2	
Yes										14.7	7.5		7.1	9.2		21.4	4.8	

*p*-values from Pearson’s Chi-Square test, statistically significant values are underlined and marked with symbols *p<0.05, **p≤0.01, and ***p≤0.001. Percentages of breast carcinomas presented according to membranous (−m), cytoplasmic (−c), and total (−t) HER3 expression; −/+ means low/high HER3 expression by IHC

In a cohort of 177 *HER2*-amplified breast carcinomas, low HER3 expression was related to clinicopathological characteristics known to predict poor clinical outcome, with the exception of the cell proliferation marker Ki67, which was not shown to associate with HER3 (Table 5). Low membranous HER3 expression was associated with negative ER status (*p* = 0.013). Low cytoplasmic HER3 expression, in turn, was related to large tumour size (≥2 cm, *p* = 0.014 or pT3-pT4, *p* = 0.001), young patient age (< 50 years) at diagnosis (*p* = 0.000), and premenopausal status (*p* = 0.000). Carcinomas with low total cellular HER3 expression were associated with lymph nodal infiltration (*p* = 0.035), cytokeratin proteins 5 and 14 expression (*p* = 0.006), and basal phenotype (*p* = 0.001). Basal phenotype was determined by concurrent cytokeratin 5/14 expression and negative ER status [87].

For NEDD4–1 and NRDP1, we found few clinicopathological correlations (Table 6). High NEDD4–1 expression was shown to correlate with high expression of the cell membrane-located HER3 protein (*p* = 0.002). The majority (87.4%, 104 of 119) of carcinomas showing high membranous HER3 expression were demonstrated to co-overexpress NEDD4–1 protein. In a group of carcinomas with low membranous HER3 expression, NEDD4–1 was negative in 38.5% (10 of 26) of carcinomas. High cytoplasmic NRDP1 expression was observed mainly in PR-positive breast carcinomas (*p* = 0.006) and correlated with total HER3 expression (*p* = 0.041). Low nuclear NRDP1 expression was observed mostly in carcinomas diagnosed in patients aged ≥50 years (*p* = 0.004). Neither nuclear nor cytoplasmic NRDP1 protein expression was associated with NEDD4–1.

**Table 6:** Associations between NEDD4–1 and NRDP1 protein expression and clinicopathological characteristics in *HER2*-amplified breast cancer cohort

Characteristic		Cytoplasmic NRDP1 expression *n* (%)			Nuclear NRDP1 expression *n* (%)			Cellular NEDD4–1 expression *n* (%)		
	*n*	NRDP1-	NRDP1+	*p*	NRDP1-	NRDP1+	*p*	NEDD4–1-	NEDD4–1+	*p*
Cases	145	133 (91.7)	12 (8.3)		133 (91.7)	12 (8.3)		25 (17.2)	120 (82.8)	
Estrogen receptor				0.057			0.206			0.421
Positive	97	86 (64.7)	11 (91.7)		87 (65.4)	10 (83.3)		15 (60.0)	82 (68.3)	
Negative	48	47 (35.3)	1 (8.3)		46 (34.6)	2 (16.7)		10 (40.0)	38 (31.7)	
Progesterone receptor				0.006**			0.125			0.053
Positive	66	56 (42.1)	10 (83.3)		58 (43.6)	8 (66.7)		7 (28.0)	59 (49.2)	
Negative	79	77 (57.9)	2 (16.7)		75 (56.4)	4 (33.3)		18 (72.0)	61 (50.8)	
Histological grade				0.953			0.446			0.134
I-II	35	32 (24.2)	3 (25.0)		31 (23.5)	4 (33.3)		9 (36.0)	26 (21.8)	
III	109	100 (75.8)	9 (75.0)		101 (76.5)	8 (66.7)		16 (64.0)	93 (78.2)	
Ki-67 proliferation index				0.228			0.228			1.000
Low	29	25 (18.8)	4 (33.3)		25 (18.8)	4 (33.3)		5 (25.0)	24 (20.0)	
High	116	108 (81.2)	8 (66.7)		108 (81.2)	8 (66.7)		20 (75.0)	96 (80.0)	
Histological type				0.022*			0.880			0.403
Ductal	128	119 (93.7)	9 (75.0)		118 (92.2)	10 (90.9)		22 (88.0)	106 (93.0)	
Lobular	11	8 (6.3)	3 (25.0)		10 (7.8)	1 (9.1)		3 (12.0)	8 (7.0)	
Lymph nodal status				0.120			0.277			0.516
Positive pN+	60	58 (45.3)	2 (20.0)		53 (42.1)	7 (58.3)		9 (37.5)	51 (44.7)	
Negative pN0	78	70 (54.7)	8 (80.0)		73 (57.9)	5 (41.7)		15 (62.5)	63 (55.3)	
Tumor size (cm)				0.669			0.820			0.715
< 2 cm	57	52 (48.1)	5 (55.6)		52 (49.1)	5 (45.5)		9 (45.0)	48 (49.5)	
≥ 2 cm	60	56 (51.9)	4 (44.4)		54 (50.9)	6 (54.5)		11 (55.0)	49 (50.5)	
Tumor size (TNM stage)				0.511			0.341			0.177
pT1-pT2	138	127 (96.2)	11 (100.0)		127 (96.9)	11 (91.7)		23 (92.0)	115 (97.5)	
pT3-pT4	5	5 (3.8)	0 (0.0)		4 (3.1)	1 (8.3)		2 (8.0)	3 (2.5)	
Patient age at diagnosis				0.856			0.004**			0.771
< 50 years	27	25 (18.8)	2 (16.7)		21 (15.8)	6 (50.0)		4 (16.0)	23 (19.2)	
≥ 50 years	118	108 (81.2)	10 (83.3)		112 (84.2)	6 (50.0)		21 (84.0)	97 (80.8)	
HER3 membrane expression				0.905			0.505			0.002**
Low	26	24 (18.0)	2 (16.7)		23 (17.3)	3 (25.0)		10 (40.0)	16 (13.3)	
High	119	109 (82.0)	10 (83.3)		110 (82.7)	9 (75.0)		15 (60.0)	104 (86.7)	
HER3 cytoplasmic expression				0.300			0.215			0.360
Low	11	11 (8.3)	0 (0.0)		9 (6.8)	2 (16.7)		3 (12.0)	8 (6.7)	
High	134	122 (91.7)	12 (100.0)		124 (93.2)	10 (83.3)		22 (88.0)	112 (93.3)	
HER3 total cellular expression				0.041*			0.942			0.620
Low	35	35 (26.3)	0 (0.0)		32 (24.1)	3 (25.0)		7 (28.0)	28 (23.3)	
High	110	98 (73.7)	12 (100.0)		101 (75.9)	9 (75.0)		18 (72.0)	92 (76.7)	
Cytokeratin 5/14 expression				0.199			0.199			0.578
Negative	127	115 (87.8)	12 (100.0)		115 (87.8)	12 (100.0)		23 (92.0)	104 (88.1)	
Positive	16	16 (12.2)	0 (0.0)		16 (12.2)	0 (0.0)		2 (8.0)	14 (11.9)	

*p*-values were calculated using Pearson’s Chi-Square test, statistically significant values are underlined and marked with *p<0.05, **p≤0.01, and ***p≤0.001

### Prognostic implications of HER3, NEDD4–1 and NRDP1 in breast cancer

In the BCA cohort, approximately one-third (36.4%, 112 of 308) of breast carcinomas developed metastatic disease recurrence during the long-term follow-up period lasting up to 22 years (mean 10.4 years). Lymph nodal infiltration pN+ (*p* = 0.000), tumour size of pT3-pT4 (*p* = 0.009), TNBC phenotype (*p* = 0.006), histological grade III (*p* = 0.007), and PR negativity (*p* = 0.035) were shown to predict breast cancer recurrence in univariate analysis (log-rank Mantel-Cox). Of these, only lymph nodal spread was of prognostic utility (*p* = 0.002, Exp (B) 2.145) for shorter RFS in multivariate Cox regression analysis. HER3, in turn, was not associated with the clinical outcome of breast cancer.

**Fig. 4:**
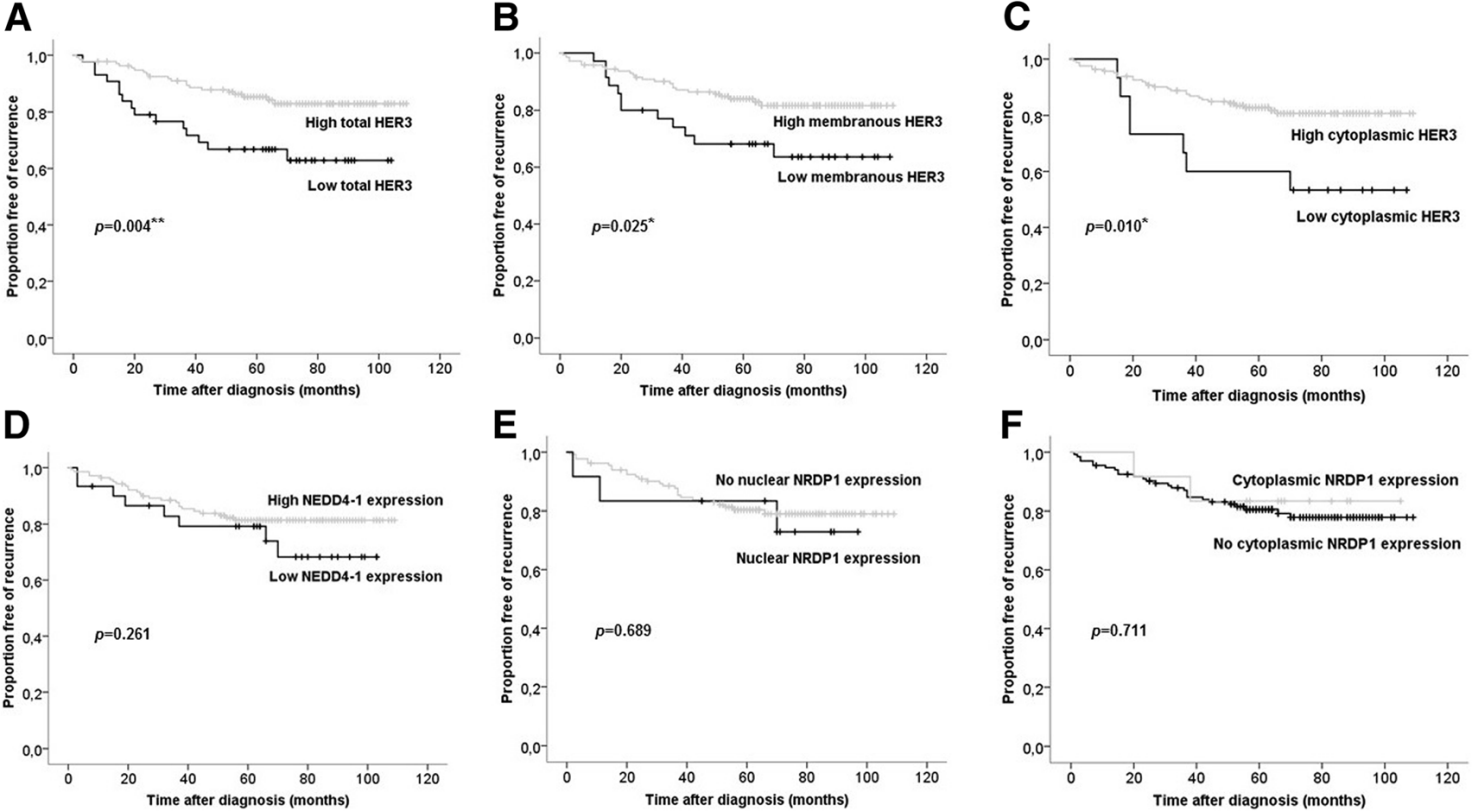
Kaplan-Meier curves showing RFS in *HER2*-amplified breast cancer (HER2+ BCA cohort) in relation to expression of **a** total cellular HER3 (*n* = 177), **b** membranous HER3 (*n* = 177), **c** cytoplasmic HER3 (*n* = 177), **d** NEDD4–1 (*n* = 145), **e** nuclear NRDP1 (*n* = 145), and F. cytoplasmic NRDP1 (*n *= 145). Log rank (Mantel-Cox) *p*-values are marked within the curves. *p*-values < 0.05 were considered statistically significant and were marked with *p<0.05 and **p≤0.01

During the mean follow-up time of 5.3 years (range 1 month to 9 years), 20.3% (36 of 177) of *HER2*-amplified breast cancer cases experienced recurrence of the disease. Distantly located metastases (61.1%, 22 of 36) were more common than local relapses (38.9%, 14 of 36). Altogether, 18.3% of patients receiving adjuvant trastuzumab therapy experienced relapse, while 22.1% of patients treated without trastuzumab were relapsing during the follow-up (*p* = 0.573). According to the univariate log-rank analysis, we found lymph nodal infiltration (*p* = 0.000), tumour size of pT3-pT4 (*p* = 0.000), and low total cellular HER3 protein expression (*p* = 0.004) as strong indicators of shortened RFS in *HER2*-amplified breast cancer (Table 7, Fig. 4). The estimated mean RFS time was shortened as follows: RFS for pN+ (vs. pN0) carcinomas was 6.7 (8.4) years, for pT3-pT4 -sized tumours (vs. pT1-pT2) 4.2 (7.9) years, and for low (vs. high) total HER3 expressing carcinomas 6.3 (8.0) years. We also found statistical significance for low membranous (*p* = 0.025) and cytoplasmic (*p* = 0.010) HER3 expression in predicting breast cancer recurrence during the follow-up period (Table 7, Fig. 4). Low total cellular HER3 expression was demonstrated to find relapsing *HER2*-amplified breast carcinomas most efficiently; 41.7% (15 of 36) of cases with recurrence were shown to demonstrate low total cellular HER3 expression. Correspondingly, one-third of relapsing carcinomas (33.3%, 12 of 36) were classified as low for membranous HER3 expression, and one-fifth (19.4%, 7 of 36) were classified as low for cytoplasmic HER3 expression. When survival analyses were performed and stratified according to adjuvant trastuzumab therapy, we observed that low total cellular and cytoplasmic HER3 expression were of prognostic utility only in a cohort treated without adjuvant trastuzumab. Based on that data, we do not see HER3 as a useful biomarker to predict the effectiveness of adjuvant trastuzumab, at least when complied with the 9-wk regimen represented in a fraction of patients in the HER2+ BCA cohort.

**Table 7:** Univariate and multivariate Cox regression analysis for prognostic value of study variables to predict RFS in *HER2*-amplified breast cancer

Characteristic	Univariate analysis Multivariate analysis						
	*n*	*p*	Mean RFS	95% CI for RFS	*p*	Exp (B)	95% CI for Exp (B)
Estrogen receptor status	177	0.090					
Progesterone receptor status	177	0.176					
Histological grade	174	0.831					
Ki-67 proliferation index	177	0.171					
Histological type (lobular/ductal)	168	0.774					
Lymph nodal status pN+ (vs pN0)	169	0.000***	6.7 (8.4)	5.9 (8.0) – 7.5 (8.7)	0.002**	3.486	1.608, 7.555
Tumor size TNM stage ≥ pT3 (vs < pT3)	172	0.000***	4.2 (7.9)	2.3 (7.5) – 6.2 (8.3)	0.001***	4.016	1.703, 9.468
Patient age at diagnosis	177	0.118					
Menopausal status	176	0.082					
Cytokeratin 5/14 expression	167	0.447					
Basal phenotype (CK5/14+, ER-)	167	0.955					
Total cellular HER3 low (vs high)	177	0.004**	6.3 (8.0)	5.3 (7.6) – 7.3 (8.4)	0.020*	2.305	1.143, 4.648
Membranous HER3 low (vs high)	177	0.025*	6.6 (7.9)	5.6 (7.4) – 7.7 (8.3)			
Cytoplasmic HER3 low (vs high)	177	0.010*	5.9 (7.8)	4.2 (7.4) – 7.6 (8.2)			
NEDD4–1 expression	145	0.261					
NRDP1 nuclear expression	145	0.689					
NRDP1 cytoplasmic expression	145	0.711					

Significant p-value (marked as *p<0.05, **p≤0.01, ***p≤0.001) means prognostic value of the variable to predict shorter RFS-time. Mean follow-up period for HER2+ BCA cohort was 5.3 years. Estimated mean RFS time is announced in years for each significant character

Based on univariate analyses, lymph nodal involvement (pN+), tumour size of pT3-pT4 and low total cellular HER3 expression were consequently tested for their prognostic value in multivariate Cox regression analysis. All of these categorized variables were independent negative prognostic factors of *HER2*-amplified breast cancer. Low total cellular HER3 protein expression was shown to increase the risk of breast cancer recurrence by 2.3-fold relapse risk, positive lymph nodal status 3.5-fold, and tumour size of pT3-pT4 by 4.0-fold (Table 7).

NEDD4–1 and NRDP1 expression did not show any prognostic value for predicting the outcome of *HER2*-amplified breast cancer in terms of recurrence-free survival (Fig. 4). Additionally, neither NEDD4–1 nor NRDP1 expression was predictive of the efficiency of short-term (9-wk schema) adjuvant trastuzumab therapy.

## Discussion

The role of HER3 in breast cancer biology has been extensively studied, especially in the context of personalized cancer therapy [1]. The current study confirmed the predominance of HER3 protein expression in primary breast cancer, as detected by IHC. The majority (75%) of breast carcinomas were shown to display intense HER3 expression irrespective of HER2 status. From a therapeutic perspective, this provides a rationale for HER3-targeted pharmaceuticals, which are defining the state of the art in breast cancer therapy, especially for *HER2*-amplified subtype. The role of anti-HER3 therapy in the treatment of HER3-dependent, non-*HER2*-amplified breast carcinomas has also been speculated recently [88]. However, e.g. lumretuzumab, in combination with pertuzumab and paclitaxel, was not confirmed clinically relevant therapy for patients with HER3-positive, HER2-low breast cancer [89], although was demonstrated effective in HER2-low/ER+ mouse xenograft model in vivo when combined with pertuzumab and endocrine (fulvestrant) therapy [90].

Interestingly, we found that low HER3 expression was associated with features that commonly define breast cancer aggressiveness: large size (≥pT3), axillary lymph nodal infiltration (pN+), negative ER status, triple-negativity (ER-, PR-, HER2-) and basal phenotype (CK5/14+, ER-). However, we were not able to find a statistically significant association between low HER3 expression and high proliferation activity (indicated by the Ki-67 proliferation index), which supports the recently published result by Takada et al. [91]. On the contrary, Kirouac et al. [92] reported earlier that HER2-positive breast cancer cells showing lower proliferation activity in vitro have concomitantly higher HER3 expression levels.

Our results demonstrate that low HER3 protein expression is indicative of shorter RFS in *HER2*-amplified breast carcinomas. Negative or low HER3 status was shown to independently increase the risk of breast cancer recurrence by two-fold. In the multivariate analysis, low membranous HER3 and low total cellular HER3 expression were prognostic factors for relapse occurrence, with well-known poor outcome determinants lymph nodal infiltration (pN+) and large tumour size (≥pT3). Despite extensive research focusing on HER3 over the past twenty years, its clinical utility in cancer prognostics - specifically in breast cancer - remains undefined [93], as has been reviewed within the current study (Table 1). When focusing on breast cancer, there are studies linking HER3 overexpression to unfavourable outcome, and others, such as the current study, that adversely associate low HER3 (mRNA or protein) expression with worse prognosis. However, some studies did not find any association between HER3 and breast cancer outcome. In addition, only some of the studies have focused on the *HER2*-amplified breast cancer subtype, in which HER signalling is specifically different from other subtypes [7]. Considering survival data, one should remember that the pattern of recurrence is already dependent on the intrinsic subtype [94], which for we have inspected our results stratified for HER2 status.

One explanation to elucidate the HER3 survival context in *HER2*-amplified breast cancer subtype could be related to intensified HER2 signalling because of paradoxical HER2 homodimerization in carcinomas with concurrently low HER3 but high HER2 expression due to amplified *HER2*. It has been previously confirmed that HER2 homodimerization is frequent, especially in breast carcinomas characterized by *HER2* gene amplification, and is related to reduced RFS [17]. In the present study, we did not find any survival differences when HER2-negative breast carcinomas (BCA cohort) with normal HER2 signalling were stratified for HER3. Earlier studies [15, 37] support that patients having both high HER2 and HER3 expression have significantly longer time to disease progression compared to patients having either high HER2 or HER3 expression in their carcinomas. Based on these observations, HER3 cannot be considered an independent prognostic factor in breast cancer overall because its clinical impact is mostly dependent on the co-expression of other HER receptors, such as HER2. Accordingly, we suggest that the HER2-HER3 interaction and its effects on growth-promoting signalling in HER2-dependent carcinomas are biologically different from carcinomas with low HER2 expression. For this reason, the prognostic applicability of HER3 should be analysed separately in breast cancers stratified for HER2 status. Additional intrinsic factors, such as the absence of HRG in HER3-overexpressing carcinomas, may also explain the finding of favourable outcomes in carcinomas characterized by high HER3 protein expression.

HER3 activation is suggested as one mechanism to account for inherent or acquired resistance to anti-HER2 therapies [19, 31, 55, 56]. The high presence of HER3 mRNA has been related to a better prognosis in patients carrying HER2-positive breast carcinoma treated with adjuvant pertuzumab therapy [38]. HER3 protein overexpression, for its part, has been shown to predict poor outcome in a group of HER2-positive breast cancer patients receiving adjuvant trastuzumab as a first-line therapy [25, 29]. In contrast, a recently published study [44] postulates that HER3 is not an informative biomarker to predict trastuzumab sensitivity. Overall, it seems that the expression profile of any single HER protein, in addition to HER2, is insufficient to predict the trastuzumab response. This is due to a complicated signalling network involving interacting HER receptors, their ligands and downstream signalling proteins [38, 49, 95].

We also analysed HER3 expression and RFS in a subgroup of patients who received adjuvant trastuzumab therapy. In the current study, HER3 expression was not shown to be predictive for adjuvant short-term (9-wk regimen) trastuzumab therapy as a first-line therapy. The recurrence rate and relapse-free survival time during the follow-up were not markedly different when stratified according to adjuvant trastuzumab therapy. Presently, one year is the recommended standard for trastuzumab therapy duration, which is based on clinical proof of prolonged survival compared to a shorter administration regimen [96–98]. This may have affected the observed recurrences in HER2+ BCA cohort, and is considered as a limitation of this study when applying these results in the current clinical practice.

The expression of HER3 receptors differs specifically from its close relative HER2. Unlike HER3, HER2 tightly attaches to the cell membrane when trafficked from the Golgi apparatus to its putative membranous location, remaining there for prolonged periods [99, 100]. This enables reliable detection and localization of HER2 protein by IHC. In contrast, HER3 receptors are unstable and constitutively internalized from the cell membrane into the cytoplasm and nucleus [101–103], which complicates the detection of this receptor type by IHC. Once internalized, HER3 is quickly ubiquitinated and transferred to proteasomes for degradation. Due to the continuous trafficking of HER3 receptors, the appearance of membrane-bound HER3 receptors does not necessarily conform the efficiency of HER3 protein synthesis machinery at the transcriptional level. There are many mechanisms in distinct facets of HER3 protein synthesis that can be disabled when HER3 is down- or up-regulated [13]. In addition, abnormal cellular HER3 receptor quantity or localization may be due to altered HER3 degradation mechanisms or the presence of exogenous stimuli with regulatory capacity on HER3 [102, 104, 105].

In the current study, we also demonstrated the expression of two regulatory proteins, NEDD4–1 and NRDP1, both of each contribute to the maintenance of HER3 receptors by mediating the degradation process via ubiquitination. We demonstrated that NEDD4–1 protein was predominantly over-expressed in *HER2*-amplified breast carcinomas; herein, 83% of carcinomas were positive for NEDD4–1. Only one earlier study clarified the NEDD4–1 protein expression pattern in breast cancer and demonstrated NEDD4–1 expression in 55% of studied cases [74]. This earlier finding is not fully comparable with the current result because of the minor representation of HER2-positive breast carcinomas. To the best of our knowledge, this is the first study to clarify the relationship between HER3 and NEDD4–1 proteins in primary breast cancer tissue in situ. In contrast to our expectation from the theoretical perspective [63], HER3 protein expression was not negatively associated with NEDD4–1 expression. In fact, we found a statistically significant parallel correlation between membranous HER3 and NEDD4–1 expression. Based on our data, we hypothesize that HER3 trafficking out from the cell membrane preceding its degradation is under more complicated controlling mechanisms than NEDD4–1 expression alone.

NRDP1 protein expression was infrequent and did not show any clinically meaningful correlations or prognostic potential to predict the outcome of *HER2*-amplified breast cancer. The absence of cytoplasmic NRDP1 expression was more common in carcinomas characterized by low cellular HER3 expression but was not otherwise associated with HER3. However, only 8.3% of carcinomas in all were shown to display nuclear or cytoplasmic NRDP1 protein expression in our *HER2*-amplified breast cancer cohort. Consequently, frequent HER3 expression in HER2-dependent breast cancer subtype does not seem to inversely associate with NRDP1 expression, but the result needs to be confirmed in a larger sample cohort because of relatively low NRDP1 expression observed in the current study. We speculate that low NRDP1 expression in *HER2*-amplified breast cancers could be mechanistically explained by the previous study of Yen et al. [75], in which NRDP1 loss was shown to enhance HER2/HER3-dependent breast tumour cell growth and tumour progression. We found only one earlier study focusing on NRDP1 expression in clinical breast cancer cohort. In this study [76], absent or low NRDP1 protein expression (approximately 42% of carcinomas) was related to worse breast cancer outcome during the ten-year follow-up period. NRDP1 expression was shown more common (approximately 58% of carcinomas) than we indicated in the current study. Comparable criteria for determining the NRDP1 expression was applied in both studies, but the IHC staining procedures and sample cohort characteristics, especially for HER2 status, were not similar and may explain the difference.

To further clarify the biological and prognostic relevance of HER3 in the therapy context of *HER2*-amplified breast cancer, many continuing research objects seem necessary. The determination of HER3 expression in metastatic lesions of breast carcinomas treated with anti-HER2 therapy, such as trastuzumab, would elucidate the concept of intensified HER3 signalling due to HER2 downregulation. HER3 upregulation has been related to trastuzumab resistance in studies [19, 106] showing that breast cancers driven primarily by HER2 homodimerization are more susceptible to trastuzumab therapy than tumours with a predominance of HER2-HER3 heterodimers. From this context, it would be interesting to determine HER3 expression in breast carcinomas that are confirmed intrinsically resistant to trastuzumab. To elucidate the therapeutic predictive potential of HER3, one intriguing thought is to clarify HER3 expression retrospectively in breast cancer patients who were subsequently treated with adjuvant pertuzumab or novel HER3-targeting antibodies.

## Conclusions

The results of the current study suggest HER3 as a novel versatile biomarker to predict recurrence of *HER2*-amplified breast cancer. Irrespective of its subcellular localization, absent or low HER3 expression was associated with shorter RFS time when compared to HER3-overexpressing breast carcinomas. Low HER3 expression was associated with clinicopathological characteristics related to more aggressive and therapeutically unfavourable breast cancer types, such as axillary lymph nodal infiltration, larger tumour size, young patient age, negative ER status, triple-negative subtype, and basal phenotype. HER3 did not show any predictive value for the benefit of short-term (9-wk) adjuvant trastuzumab therapy as a first-line therapy. The HER3 degradation regulators NEDD4–1 and NRDP1 did not show any clinically meaningful correlations or predictive or prognostic applicability in *HER2*-amplified breast cancer subtype.

## Additional file

Additional file 1: IHC-staining protocols provide detailed information on reagents used in the current study to demonstrate HER3, NEDD4–1, NRDP1, and Cytokeratin 5/14 protein expression on FFPE breast cancer tissues. (PDF 283 kb)

## Abbreviations

ER: Oestrogen receptor
FFPE: Formalin-fixed paraffin-embedded tissue
FLRF: Fetal liver ring finger
HER1/2/3: Human epidermal growth factor receptor 1/2/3
HIER: Heat-induced epitope retrieval
HRG: Heregulin
IHC: Immunohistochemistry
NEDD4–1: Neural precursor cell expressed developmentally downregulated 4–1
NRDP1: Neuregulin receptor degradation protein-1
PR: Progesterone receptor
RFS: Recurrence free survival
RNF41: RING finger protein 41
ROI: Region of interest
TMA: Tissue microarray
TNBC: Triple-negative breast cancer
