# Supplementary material for: Clinicopathological and prognostic correlations of HER3 expression and its degradation regulators, NEDD4–1 and NRDP1, in primary breast cancer
Source: BMC Cancer. 2018 Oct 26;18:1045. doi: 10.1186/s12885-018-4917-1 (PMC6204010; doi:10.1186/s12885-018-4917-1)
Supplement: Supplementary file 1 — IHC-staining protocols provide detailed information on reagents used in the current study to demonstrate HER3, NEDD4–1, NRDP1, and Cytokeratin 5/14 protein expression on FFPE breast cancer tissues. (PDF 283 kb) [file 12885_2018_4917_MOESM1_ESM.pdf]

Additional file 1. IHC-staining protocols used in the current study for demonstrating HER3, NEDD4-1, NRDP1, and Cytokeratin 5/14 protein expression on FFPE breast cancer tissue (LabVision Autostainer platform).

**HER3 IHC**	**NEDD4-1 IHC**	**NRDP1 IHC**	**Cytokeratin 5/14 IHC**
**Pretreatments**: deparaffinization, HIER in Tris-EDTA (TE) buffer pH9 +98°C 15 min<br>Rinse with TBS-Tween (0.05%) × 1<br>**Step 1**: *Protein Blocking*[1] 5 min<br>Rinse with TBS-Tween (0.05%) × 1<br>**Step 2**: *HER3 primary antibody*[2] 1:100 30 min<br>Rinse with TBS-Tween (0.05%) × 2<br>**Step 3**: *Post-antibody blocking*[3] 20 min<br>Rinse with TBS-Tween (0.05%) × 2<br>**Step 4**: *HRP-conjugated secondary antibody*[4] 30 min<br>Rinse with TBS-Tween (0.05%) × 2<br>**Step 5**: *3,3'-Diaminobenzidine (DAB)*[5] 5 min<br>Rinse with distilled water × 1 and TBS-Tween (0.05%) × 2<br>**Step 6**: *Mayer's Hematoxylin* 2 min<br>Rinse with distilled water × 1 and TBS-Tween (0.05%) × 1	**Pretreatments**: deparaffinization, HIER in Tris-EDTA (TE) buffer pH9 +98°C 15 min<br>Rinse with TBS-Tween (0.05%) × 1<br>**Step 1**: *3% Hydrogen Peroxide (H_2O_2)* 5 min<br>Rinse with TBS-Tween (0.05%) × 1<br>**Step 2**: *NEDD4-1 primary antibody*[1] 1:750 30 min<br>Rinse with TBS-Tween (0.05%) × 2<br>**Step 3**: *Post antibody blocking*[2] 20 min<br>Rinse with TBS-Tween (0.05%) × 2<br>**Step 4:** *HRP-conjugated secondary antibody*[3] 30 min<br>Rinse with TBS-Tween (0.05%) × 2<br>**Step 5**: *3,3'-Diaminobenzidine (DAB)*[4] 5 min<br>Rinse with distilled water × 2 and TBS-Tween (0.05%) × 1<br>**Step 6**: *0.5% Copper sulfate ($CuSO_4$)* added as enhancement in *Mayer's Hematoxylin* 5 min<br>Rinse with distilled water × 1 and TBS-Tween (0.05%) × 1	**Pretreatments**: deparaffinization, HIER in Tris-EDTA (TE) buffer pH9 +98°C 20 min<br>Rinse with TBS-Tween (0.05%) × 1<br>**Step 1**: *FLRF/RNF41 primary antibody*[1] 1:3000 30 min<br>Rinse with TBS-Tween (0.05%) × 2<br>**Step 2**: *3% Hydrogen Peroxide (H_2O_2)* 5 min<br>Rinse with TBS-Tween (0.05%) × 2<br>**Step 3**: *HRP-conjugated secondary antibody*[2] 30 min<br>Rinse with TBS-Tween (0.05%) × 2<br>**Step 4:** *3,3'-Diaminobenzidine (DAB)*[3] 10 min<br>Rinse with distilled water × 1 and TBS-Tween (0.05%) × 1<br>**Step 5**: *0.5% Copper sulfate ($CuSO_4$)* enhancement 5 min<br>Rinse with TBS-Tween (0.05%) × 2<br>**Step 6**: *Mayer's Hematoxylin* 1:2<br>Rinse with distilled water × 1 and TBS-Tween (0.05%) × 1	**Pretreatments**: deparaffinization, HIER in Tris-EDTA (TE) buffer pH9 +98°C 15 min<br>Rinse with TBS-Tween (0.05%) × 1<br>**Step 1**: *3% Hydrogen Peroxide (H_2O_2)* 5 min<br>Rinse with TBS-Tween (0.05%) × 1<br>**Step 2**: *CK 5/14 primary antibody*[1] *cocktail* 1:150 30 min<br>Rinse with TBS-Tween (0.05%) × 2<br>**Step 3**: *Post antibody blocking*[2] 20 min<br>Rinse with TBS-Tween (0.05%) × 2<br>**Step 4:** *HRP-conjugated secondary antibody*[3] 30 min<br>Rinse with TBS-Tween (0.05%) × 2<br>**Step 5**: *3,3'-Diaminobenzidine (DAB)*[4] 5 min<br>Rinse with distilled water × 2 and TBS-Tween (0.05%) × 1<br>**Step 6**: *Mayer's Hematoxylin* 2 min<br>Rinse with distilled water × 1 and TBS-Tween (0.05%) × 1
Additional reagent information:<br><br>1) Ultra Vision Protein Block (LabVision),<br>2) Mouse monoclonal HER3 antibody DAK-H3-IC (Dako, M7297) diluted in Normal Antibody Diluent (ImmunoLogic, BD09-999),<br>3) BrightVision Plus Post-antibody blocking<br>4) BrightVision Plus Poly-HRP-Anti Ms/Rb IgG (ImmunoLogic, DPVB110 HRP),<br>5) ImmPACT™ DAB Peroxidase Substrate, dilution: 25 µl of Chromogen concentrate added to each 1 ml of DAB Diluent (Vector Laboratories Inc., SK-4105)	*Additional reagent information*:<br><br>1) Rabbit polyclonal Anti-Nedd4 antibody (Merck KGaA, #07-049) diluted in Normal Antibody Diluent (ImmunoLogic, BD09-999),<br>2) BrightVision Plus Post-antibody blocking,<br>3) BrightVision Plus Poly-HRP-Anti Ms/Rb IgG (ImmunoLogic, DPVB110 HRP),<br>4) Histofine® DAB-2V, dilution: 40 µl Chromogen reagent added to each 1 ml of Chromogen substrate (Nichirei Biosciences Inc., 425312F)	*Additional reagent information*:<br><br>1) Rabbit polyclonal FLRF/RNF41 antibody (Bethyl Laboratories Inc., A300-049A) diluted in Normal Antibody Diluent (ImmunoLogic, BD09-999),<br>2) EnVision™ FLEX High pH HRP,<br>3) EnVision™ FLEX DAB+, dilution: 50 µl DAB+ Chromogen added to each 1 ml of DAB+ Substrate Buffer (Dako, K8010)	*Additional reagent information*:<br><br>1) Mouse monoclonal antibodies for CK5, clone NCL-L-CK5 (Leica Biosystems, XM26), and CK14, clone NCL-L-LL0022 (Leica Biosystems, LL0022), diluted in Normal Antibody Diluent (ImmunoLogic, BD09-999),<br>2) BrightVision Plus Post-antibody blocking,<br>3) BrightVision Plus Poly-HRP-Anti Ms/Rb IgG (ImmunoLogic, DPVB110 HRP),<br>4) ImmPACT™ DAB Peroxidase Substrate, dilution: 25 µl of Chromogen concentrate added to each 1 ml of DAB Diluent (Vector Laboratories Inc., SK-4105)
